# Project interpret cholangiogram at the SAGES 2024 Education & Innovation Center: skill deficiency in bile duct injury recognition among surgical residents

**DOI:** 10.1007/s00464-025-12145-x

**Published:** 2025-09-13

**Authors:** Emily M. Hannah, Matthew B. Bloom, Steven D. Schwaitzberg

**Affiliations:** 1 Jacobs School of Medicine and Biomedical Sciences, University at Buffalo, Buffalo, NY USA; 2 Department of Surgery, Cedars Sinai Medical Center, Los Angeles, CA USA; 3 Department of Surgery, University at Buffalo, Buffalo, NY USA; 4 Department of Surgery, Buffalo General Hospital, 100 High Street #D-3, Buffalo, NY 14203 USA

**Keywords:** Laparoscopic cholecystectomy, Intraoperative cholangiography (IOC), Medical education, Confidence, Resident training

## Abstract

**Background:**

Bile duct injuries (BDI) remain a rare but dreaded complication of laparoscopic cholecystectomy (LC). Proposed solutions to reduce BDI involve increased use of cholangiograms in patients with ambiguous anatomy. One explanation for the underutilization of intraoperative cholangiograms (IOC) is an unfamiliarity in interpreting cholangiograms, particularly amongst surgical trainees. We aim to gauge the current confidence levels and knowledge state of interpreting cholangiograms of surgeons at all levels of training.

**Methods:**

Participants were screened based on their SAGES 2024 Annual Conference attendance and completed the “Cholangiogram Quiz” at a station in the SAGES Education & Innovation Center. Data were collected on training background, and confidence in identifying IOC findings. Participants (*n* = 88, surgical residents, fellows and attendings) completed eighteen multiple choice questions on interpreting a cholangiogram.

**Results:**

Overall quiz scores and confidence in interpreting IOC increased with level of training (*p* < 0.001). Out of normal IOC, aberrant ducts, BDI, common bile duct calculi, and filling defects, PGY1-5 residents were least confident identifying aberrant ducts, followed by BDI. PGY1-3 residents performed worse than attendings on questions on BDI (*p* < 0.001). Differences in quiz performance by training levels did not reach statistical significance for any other topic tested. Better quiz performance was directly correlated to more frequent use of IOC (*p* < 0.001). Higher confidence was associated with better quiz performance for all participants (*p* = 0.006). Confidence interpreting normal IOC and BDI on cholangiogram were directly correlated to quiz performance on these topics (normal IOC: *p* = 0.005, BDI: *p* = 0.047).

**Conclusions:**

Participants with more advanced training, and who more frequently utilize IOC performed better on our quiz. Compared to other findings seen on IOC, residents failed to identify BDI. Surgical residents would benefit from targeted educational interventions to bolster confidence and improve accuracy in identifying BDI on IOC.

**Graphical abstract:**

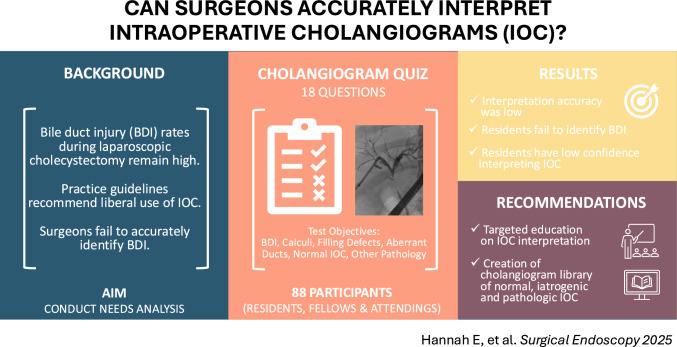

**Supplementary Information:**

The online version contains supplementary material available at 10.1007/s00464-025-12145-x.

Despite a growing body of literature supporting modest improvements in the rates of bile duct injuries (BDI) as we surpass the “learning curve” from the advent of laparoscopic cholecystectomy (LC), BDI remains a dreaded complication of LC with significant associated morbidity and mortality [1–3]. Increased rates of BDI with the introduction of LC compared to open cholecystectomy prompted the development of the Society of American Gastrointestinal and Endoscopic Surgeons Safe Cholecystectomy Program (SAGES-SCP) to promote a universal culture of safety in LC [4]. Proposed solutions to prevent and reduce incidence of BDI involve establishing the critical view of safety, opting for bailout procedures or advice from a second surgeon if the dissection becomes too dangerous or technically difficult, understanding the potential for aberrant anatomy, and increasing the utilization of cholangiograms—particularly in patients with ambiguous anatomy [4].

While some literature has found surgical inexperience was not a risk factor for BDI in LC, [5] other studies have suggested higher rates of bile duct injury occur in younger and less experienced surgeons [6]. However, as many of the tenets posed by the SAGES-SCP rely on advanced surgical experience, this finding could be explained by failure to engage young surgeons who should be capable of safely completing one of the most common procedures in general surgery. As experience cannot be circumvented, McKinley et al. noted that procedural skill is an inappropriate educational target for reduction in BDI incidence [7]. Rather, they propose targeted educational interventions with emphasis on cognitive skills and operative judgement for improving incidence of BDI [7]. Prior to the administration of an educational curriculum with these aims, it is important to delineate and address gaps in content knowledge.

Failure to perform intraoperative cholangiography (IOC) has been associated with increased risk of BDI, especially for inexperienced surgeons [8, 9]. Mechanisms by which IOC reduces the incidence of BDI will be discussed later in this study. The SAGES-SCP recommendation to employ liberal use of IOC rests on the assumption that IOC is interpreted accurately, as performance of IOC alone is insufficient to prevent or reduce BDI [4, 10, 11]. Unfamiliarity in interpreting cholangiograms, particularly among surgical residents and inexperienced surgeons, constitutes a potential knowledge deficit that warrants investigation.

This unrealized gap in education is enabled by the absence of available teaching resources on accurate cholangiogram interpretation, which would further preclude performing IOC in practice. Our project aims to conduct a needs analysis to gauge the current confidence and knowledge state of interpreting cholangiograms of surgeons at all levels of training which could inform future educational interventions aimed at BDI rate reduction.

## Methods

### Cholangiogram consortium creation

A copy of *Operative Biliary Radiology* by George Berci and J. Andrew Hamlin, a text dated from 1981 and out of print, was obtained [12]. 244 cholangiograms from the text were scanned and each cholangiogram was digitally manipulated to improve resolution and contrast using Adobe Photoshop [13]. Captions for each cholangiogram were cataloged. Eleven additional cholangiograms were de-identified and included for the quiz. The authors then reviewed all cholangiograms for suitability. Cholangiograms with insufficient quality were excluded for possible use in the cholangiogram quiz.

### Quiz development

The final cholangiogram quiz **(Appendix A)** was created to include eighteen multiple choice questions with images from the cholangiogram consortium that tasked the participant to interpret the cholangiogram image. The quiz contained a well-balanced representation of IOC findings, referred to as “test objectives.” Test objectives included normal IOC, calculi and filling defects, aberrant ducts, BDI and other pathology.

### SAGES 2024 Education & Innovation Center: testing stage

Participants were screened through a targeted recruitment process based on their attendance at the SAGES 2024 Annual Conference in Cleveland, Ohio. Participants who self-identified as a surgeon or surgical trainee (resident or fellow) with a background in General Surgery were deemed eligible to participate. The questionnaire was administered in a controlled environment through Google Forms on a computer at the SAGES 2024 Education & Innovation Center. Background data were collected on the participant’s current training level, any fellowship/specialty training, years in practice, frequency of utilization of IOC during LC over the past year, and current confidence in identifying IOC findings. Current confidence in cholangiogram interpretation was assessed via Likert scales. Cholangiogram interpretation was assessed by the eighteen-question multiple choice quiz with images from the cholangiogram consortium that met the test objectives. There was no time limit for the quiz. Upon quiz completion, participants could opt to leave their contact information to participate in an iPad raffle and/or to be notified of the correct answers upon conclusion of the conference. This study was approved by the University at Buffalo IRB (IRB ID: STUDY00007542).

### Data analysis

Participants were stratified into four groups based on current training level (PGY1-3, PGY4-5, PGY6-8/Fellow, Attending). Quiz questions were stratified into six test objectives (Table 1) for further analysis.

**Table 1:** Cholangiogram quiz stratification by test objective

Test objectives	Question number
BDI	Q1, Q12, Q18
Other pathology	Q2, Q10, Q16
Calculi	Q3, Q6, Q8, Q14
Filling defect	Q4, Q15
Aberrant ducts	Q5, Q7, Q11, Q17
Normal IOC	Q9, Q13

Individual question responses were qualified as correct or incorrect. Responses were pooled by training level, organized into 4 × 2 contingency tables and assessed using a two-tailed Fisher’s exact test in RStudio [14]. Significant results underwent post-hoc analysis to identify pairwise differences in quiz performance between training levels.

Confidence on cholangiogram interpretation by training level and test objective was assessed using ANOVA. Confidence for the test objective “other pathology” was not asked. Rate of IOC use during LC, frequency of performing LC, and confidence on cholangiogram interpretation were independently compared to quiz performance and evaluated by a two-tailed Spearman’s correlation coefficient (r_s_). Rate of reported IOC utilization and frequency of performing LC were then stratified and assigned an integer. The product of these integers is referred to as the “Gallbladder Multiplier” to denote the rate of IOC utilization as a function of frequency of performing LC. The Gallbladder Multiplier was also compared to quiz performance using two-tailed Spearman’s correlation coefficient. A *p* value < 0.05 was considered statistically significant.

## Results

### Participant training background

There were eighty-eight participants (fifty-three Residents, nine Fellows, twenty-six Attendings) who self-selected to participate. Participant training level and stratification into analysis groups can be seen in Table 2. The Postgraduate Year (PGY) 6–8/Fellow group included nine Fellows, and one Resident.

**Table 2:** Stratified groups for analysis by training level (*n* = 88)

Total Participants (88)
PGY1-3 (29)
PGY-1 (7)
PGY-2 (8)
PGY-3 (14)
PGY4-5 (23)
PGY-4 (14)
PGY-5 (9)
PGY6-8/Fellow (10)*
PGY-6 (5)
PGY-7 (1)
PGY-8 (4)
Attendings (26)
1–10 YIP (16)
> 10 YIP (10)

^*^PGY6-8/Fellow group included 1 resident

Minimally Invasive Surgery and/or Bariatric Surgery was the most common advanced training among the Fellow group (77%). One fellow indicated training in Trauma, Acute Care Surgery and Surgical Critical Care, and one fellow did not specify.

In the Attending group, 69% of participants indicated advanced training in Minimally Invasive Surgery and/or Bariatric Surgery. Three Attendings reported they had advanced training in Trauma, Acute Care Surgery, and Surgical Critical Care, two in Pediatric Surgery, one in Colorectal Surgery, and two did not specify specialized training.

### Participant IOC utilization

**Fig. 1:**
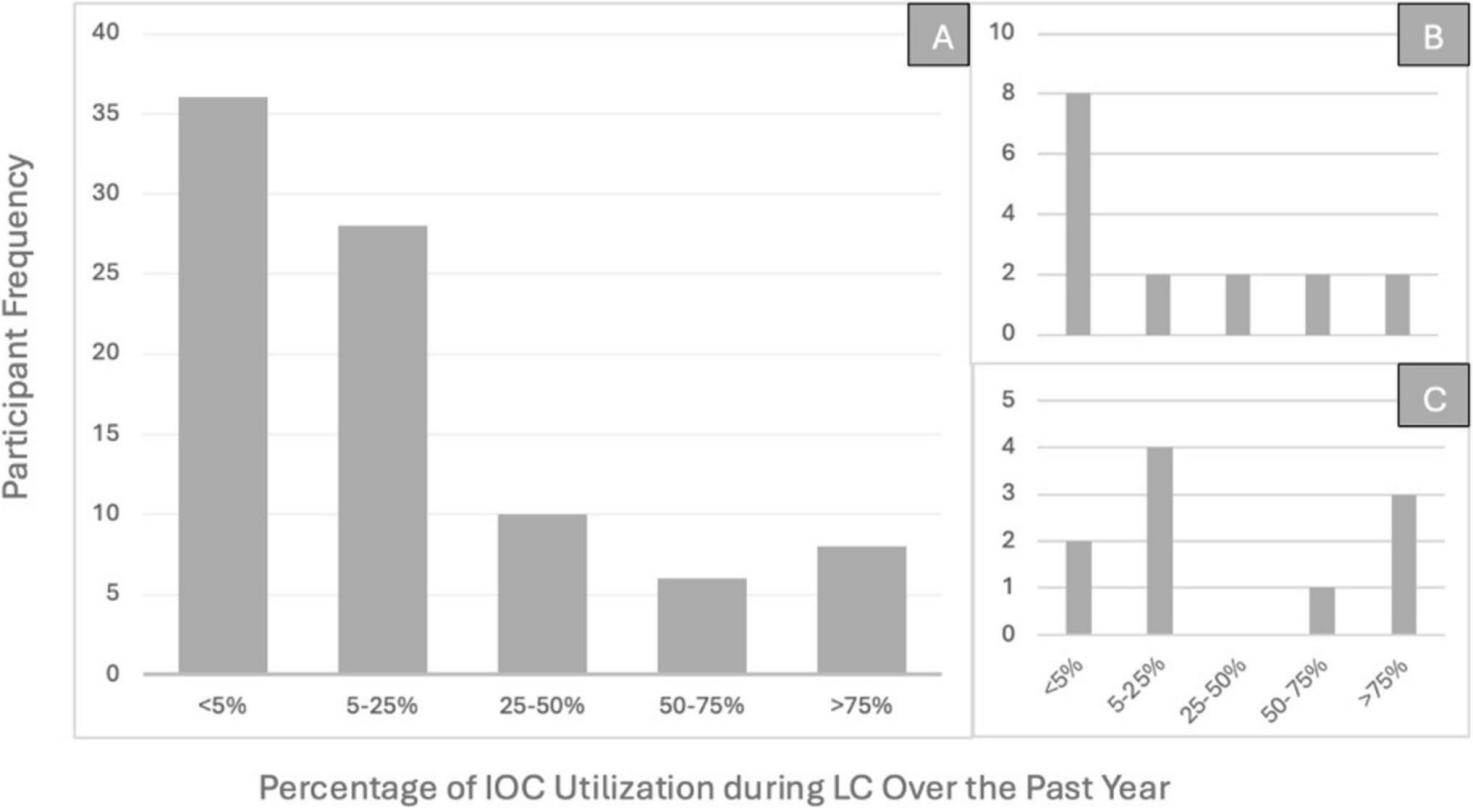
Reported utilization of IOC during LC over the past year. **A** All study participants (*n* = 88). **B** Attendings 1–10 years in practice (*n* = 16). **C** Attendings 10 + Years in Practice (*n* = 10)

Overall, reported IOC use during LC by participants in our study was low. Seventy-three percent of the participants in our study reported using IOC < 25% of the time during LC over the past year, and nine percent of used IOC > 75% of the time (Fig. 1a). Attendings 1–10 YIP (*n* = 16) in our study reported less utilization of IOC during LC than Attendings 10 + YIP (*n* = 10). Of the Attendings 1–10 YIP, 50% reported using IOC < 5% of the time during LC (Fig. 1b). The most frequent utilization of IOC for Attendings 10 + YIP (*n* = 10) was 5–25% of the time during LC (40%), followed by > 75% of the time (30%), (Fig. 1c).

### Quiz results

**Fig. 2:**
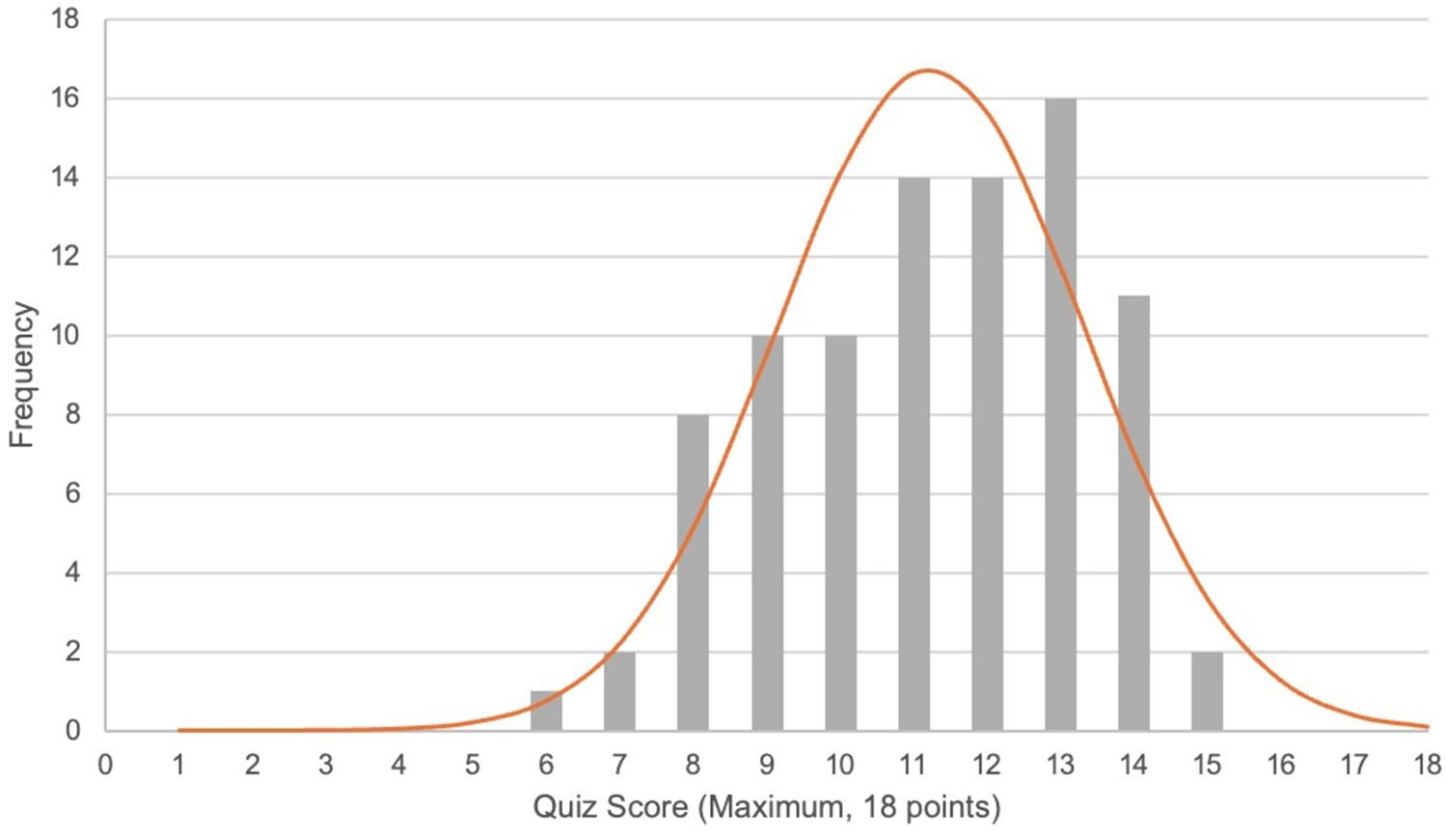
Total scores on cholangiogram quiz (*n* = 88) at SAGES 2024 Education & Innovation Center with normal distribution curve

Quiz scores were calculated out of a maximum of 18 points (range: 6–15). The mean, median and mode for the total quiz scores were 11, 11, and 13, respectively. The quiz scores approximated a normal distribution (Fig. 2).

Increased frequency of performing LC had a weak, positive correlation (*r*_*s*_: + 0.070) with overall quiz performance, this relationship did not reach statistical significance (*p* = 0.515). More frequent use of IOC was positively correlated (*r*_*s*_: + 0.394) with better quiz performance (*p* < 0.001). A higher Gallbladder Multiplier, or a higher rate of IOC use as a function of frequency of performing LC, was positively correlated with quiz performance (*r*_*s*_: + 0.323, *p* = 0.002).

Cumulative performance on the cholangiogram quiz by each training level can be seen in Table 3. PGY1-3's had the lowest scores on the cholangiogram quiz, answering 57% of quiz questions correctly. PGY1-3’s quiz performance was significantly lower compared to the PGY6-8/Fellows (67%, *p* = 0.022), and the Attendings (68%, *p* < 0.001). PGY4-5’s answered 60% of quiz questions correctly, which was significantly lower than the Attending group (68%, *p* = 0.020). PGY6-8/Fellows had higher scores than PGY4-5’s, however, this difference did not reach statistical significance.

**Table 3:** Overall performance on cholangiogram quiz by training level

Training Level (quiz % correct)	*p *value
PGY1-3 (57%) vs. PGY 4–5 (60%)	0.350
PGY1-3 (57%) vs. PGY 6–8/F (67%)	**0.022**
PGY1-3 (57%) vs. Attendings (68%)	** < 0.001**
PGY4-5 (60%) vs. PGY 6–8/F (68%)	0.118
PGY4-5 (60%) vs. Attendings (68%)	**0.020**
PGY6-8/F (67%) vs. Attendings (68%)	0.852

Bold indicates statistically significant *p*-values.

PGY1-3 and PGY4-5’s correctly identified BDI 53% and 67% of the time. PGY6-8/Fellows answered 70% of BDI questions correctly, while attendings identified BDI 79% of the time (Table 4). Differences in performance on the BDI test objective questions underwent post-hoc analysis. When each group was compared, only performance between PGY1-3’s vs Attendings on the BDI test objective reached statistical significance (*p* < 0.001). Differences between groups did not reach statistical significance for any other test objective.

**Table 4:** Performance on cholangiogram quiz by test objective for each training level

Test Objective (% correct, n responses)	PGY1-3	PGY4-5	PGY6-8/F	Attendings	*p* value
BDI	53%, 87	67%, 69	70%, 30	79%, 78	**0.004**
Other pathology	72%, 87	65%, 69	63%, 30	69%, 78	0.698
Calculi	72%, 116	80%, 92	88%, 40	80%, 104	0.155
Filling defect	53%, 58	35%, 46	50%, 20	54%, 52	0.199
Aberrant ducts	44%, 116	50%, 92	55%, 40	60%, 104	0.130
Normal IOC	43%, 58	50%, 46	70%, 20	56%, 52	0.189

Bold indicates statistically significant *p*-values.

### Confidence in cholangiogram interpretation

Average reported confidence in interpreting cholangiograms differed amongst training levels, these differences reached statistical significance for all test objectives except for calculi (Table 5). Average overall confidence tended to increase with years of training (*p* < 0.001). On average, all training levels were least confident identifying aberrant ducts, followed by BDI.

**Table 5:** Average reported confidence by test objective for each training level

Test Objective	PGY1-3	PGY4-5	PGY6-8/F	Attendings	*p* value
BDI	5.10	6.17	7.60	7.46	** < 0.001**
Calculi	6.38	7.09	7.80	7.69	0.069
Filling defect	6.10	7.13	7.80	7.62	**0.012**
Aberrant duct	4.14	5.52	6.10	6.85	** < 0.001**
Normal IOC	6.14	7.22	7.90	8.12	**0.001**
Overall (out of 10)	5.57	6.63	7.44	7.55	** < 0.001**

Bold indicates statistically significant* p*-values.

Higher overall average confidence was correlated with better overall quiz performance (*r*_*s*_: + 0.293, *p* = 0.006) for all participants (Table 6). Increased confidence for each test objective was also positively correlated with better overall performance, this finding reached statistical significance for all topics except for calculi, which had the weakest correlation to overall quiz performance (*r*_*s*_: + 0.208, *p* = 0.052). Of the test topics, higher confidence interpreting normal IOC was the most strongly correlated to accurate interpretation of IOC on our quiz (*r*_*s*_: + 0.386, *p* < 0.001). Participants with higher confidence interpreting normal IOC and BDI, performed better on questions testing Normal IOC (*r*_*s*_: + 0.295, *p* = 0.005) and BDI (*r*_*s*_: + 0.212, *p* = 0.047), inversely, participants with lower confidence tended to perform worse on these test objectives. Confidence interpreting cholangiograms showing aberrant ducts (*r*_*s*_: + 0.101), calculi (*r*_*s*_: + 0.073), and filling defects (*r*_*s*_: + 0.075) had a weak, positive correlation with performance on these questions, and these relationships did not reach statistical significance.

**Table 6:** Relationship between participant (*n* = 88) confidence on cholangiogram quiz topic and quiz performance corresponding topic

Topic	Confidence in Interpretation (*p* value)					
	BDI	Calculi	Filling Defect	Aberrant Duct	Normal IOC	Overall
Quiz performance, by corresponding topic	r_s_: + 0.212 **(0.047)**	r_s_: + 0.073 (0.497)	r_s_: + 0.075 (0.486)	r_s_: + 0.101 (0.347)	r_s_: + 0.295 **(0.005)**	-
Quiz performance, overall	r_s_: + 0.257 **(0.016)**	r_s_: + 0.208 (0.052)	r_s_: + 0.225 **(0.035)**	r_s_: + 0.263 **(0.013)**	r_s_: + 0.386 **(< 0.001)**	r_s_: + 0.293 **(0.006)**

*r*_*s*_ Spearman’s correlation coefficient

Bold indicates statistically significant *p*-values.

Confidence for Attendings 1–10 years in practice (YIP) compared to 10 + YIP was assessed separately. Differences in confidence identifying normal IOC, aberrant ducts, BDI, calculi and filling defects was not statistically significant. There was no significant difference detected between groups for quiz performance on identifying BDI, stones, filling defects, aberrant ducts, or normal IOC. Attendings 10 + YIP performed better on questions related to other pathology (*p* = 0.011) compared to those with 1–10 YIP. Attendings with 10 + YIP more accurately identified CBD diverticula, and a malignant obstruction of CBD compared to those 1–10 YIP. Overall, attendings 1–10 YIP scored 64% on the quiz, while Attendings 10 + YIP scored 74% (*p* = 0.033).

## Discussion

To our knowledge, this is the first study to assess specific deficiencies in IOC interpretation based on surgeon experience. Overall scores on the cholangiogram quiz were low which is consistent with existing literature on cholangiogram interpretation [15]. However, the present study was designed to analyze differences in performance for each test objective based on training level. As expected, surgical trainees performed worse than attendings on all test objectives. However, relative performance of PGY1-5’s compared to attendings by test objective revealed the differences in performance only reached statistical significance for the PGY1-3 group with questions that tested recognition of BDI. Although the PGY6-8/Fellow group performed better than attendings on topics related to calculi and normal IOC, these differences were not significant. We feel limited conclusions can be drawn from the results of the PGY6-8/Fellow group due to low sample size and heterogeneity of scope of practice as a senior resident compared to that, for example, of a Bariatric Surgery Fellow.

Participants who performed IOC more frequently in clinical practice tended to score higher on the cholangiogram quiz. Attendings with over ten years of practice scored significantly higher overall. Our quiz has not been validated as a direct measure of IOC interpretation in the operating room, but these findings suggest that interpretation accuracy improves with experience and repetition. This conclusion should not be misconstrued as an argument for routine versus selective IOC as that is beyond the scope of this study. However, it is worth noting that when Sanjay et al. assessed IOC interpretation of surgical trainees and surgeons, correct interpretation was not associated with practice of routine versus selective IOC [15].

IOC utilization rates during LC vary significantly by country, institution culture, and attending preference. A systematic review by Pucher et al. reported a BDI rate of 0.32–0.52% across 106 studies and an IOC utilization rate of 5.69–26.3% across 55 studies from multiple countries [16]. Data on specific rates by country is limited, but Sweden has reported more routine use [17], while in the United States (US), IOC utilization remains inconsistent and largely depends on individual surgeon preferences since most US hospitals do not have defined policies on IOC use.

Possible explanations for the discrepancy highlighted in our study when comparing PGY1-5’s to attendings may be due to limited clinical exposure to BDI during surgical residency in the US, insufficient education on its recognition, or a component of both. For example, a surgical trainee in a 5-year surgical program graduates having participated in approximately 150 LC [18], but if this resident were from an institution with low utilization rate, they may never encounter or recognize BDI during general surgery training, let alone identify BDI intraoperatively on IOC [19]. This finding is supported by the low IOC utilization rates observed in our study, where nearly 73% of participants reported use of IOC < 5% of the time during LC over the past year, particularly when considering 59% of our participants belonged to the PGY1-5 groups. When examining IOC utilization trends during LC from 2012 to 2023 for general surgery residents in the US, Caldwell et al. found experience with IOC has declined during training and when IOC is performed, it is typically reserved for senior (PGY5) residents [19]. These findings will further complicate future discussions of defining when a surgical resident is expected to know how to perform and interpret IOC.

To discuss further implications of resident failure to identify BDI on IOC in our study, it is important to acknowledge that IOC serves two purposes related to BDI. The first of which is routine performance to identify anatomy with certainty that may be difficult to discern from dissection alone. “Shooting” a cholangiogram does not eliminate the possibility of injury to critical structures but improves the safety of LC through improved familiarity with anatomy and potential variants that are not otherwise apparent [10, 20, 21]. The second is to facilitate timely diagnosis and limit extension of injury if BDI is suspected which could lead to a reduction in BDI associated morbidity and mortality [22].

Currently, the Accreditation Council for Graduate Medical Education (ACGME) does not require general surgery residents to demonstrate competency in diagnostic radiology, including cholangiography. As a result, radiology education during general surgery training is program dependent. Aziz et al. found that 76.6% of surveyed residents reported no formal radiologic training during residency [23]. Similarly, Eid et al. reported that only 8.3% of general surgery programs in their study offered a dedicated radiology rotation for their residents, however, it remains unclear how a formal rotation affects interpretation accuracy for general surgery residents [24].

Significant differences between radiologist and surgeon IOC interpretation exist, although, this was not been associated with adverse clinical outcome [25]. It is important to note that the study that reported this finding did not report any BDI [25]. Unlike other imaging modalities, IOC interpretation in the operating room may not allow for formal review by a trained radiologist, and in some centers IOC are exclusively interpreted by the performing surgeon [23, 25]. Instead, surgeons—often without validated training on cholangiogram interpretation—must make timely and accurate assessments of anatomy and pathology, particularly when BDI is suspected.

IOC can be safely performed by resident surgeons when supervised by an expert attending surgeon who routinely performs IOC, and experienced surgeons should take the opportunity to engage residents to review their interpretation of cholangiograms [18, 26]. However, this alone cannot ensure a universal improvement in resident competency, and actual educational value is limited based on data showing a 79% failure rate of surgeons to detect BDI on IOC in practice [27]. To address this, it would be beneficial to employ standardized education, particularly for residents, demonstrating BDI in a low-stakes environment where interpretations can be verified for accuracy.

For those who do not routinely perform IOC, confidence and knowledge deficits hinder its use in identifying biliary pathology. Trainees exhibited significantly lower confidence across all gallbladder and biliary topics compared to more experienced residents and attendings. Wightkin et al. demonstrated that perceptual learning training improved surgical residents' IOC interpretation skills and confidence [28]. Similarly, our study found that confidence in identifying normal IOC and BDI correlated with better performance on related quiz questions. Through participation in a pilot online surgical education tool on IOC interpretation, Kaldas et al. found improved overall accuracy in identifying the key elements of IOC amongst their cohort of seven surgical trainees [29]. Interestingly, when examining test objective confidence in our study, confidence interpreting normal IOC was most closely correlated to overall accuracy on our quiz, even more than overall confidence levels. Future work should emphasize a high volume of normal IOC as improved confidence in this area may be a proxy for overall accuracy in cholangiogram interpretation. These findings further support that simulation-based teaching tools or educational modules could improve trainee confidence and accuracy in IOC interpretation. Interest in cholangiogram interpretation could also be fostered through topical inclusion by the American Board of Surgery through its SCORE curriculum, ABSITE examination, qualifying and certifying examinations.

Our study has several limitations. Many cholangiograms used in the quiz were outdated and enhanced using visual editing software, which was deemed necessary to approximate modern IOC resolution. Additionally, the study only included still cholangiograms. Future research should incorporate dynamic fluoroscopic cholangiograms for better translation to clinical practice. These limitations highlight the scarcity of educational cholangiograms, which remains a barrier to developing effective training tools.

IOC remains a critical skill every surgeon should possess [30] particularly as an adjunct to establishing a culture of safety in LC [4]. IOC application is hindered by deficiencies in interpretation accuracy. We hope this study encourages data sharing within the surgical community to contribute to our cholangiogram consortium and development of widespread programming on accurate IOC interpretation is imperative. For rare complications such as BDI, which can have devastating consequences, sharing diagnostic images and formal review of their findings beyond program-led morbidity and mortality conferences—such as at national and international meetings—would significantly benefit trainees, practicing surgeons and indirectly, patients. Further work should assess whether such educational interventions lead to clinically significant improvements in BDI recognition and incidence.

## Supplementary Information

Below is the link to the electronic supplementary material.

Supplementary file1 (PDF 610 KB)
