# Supplementary material for: Project interpret cholangiogram at the SAGES 2024 Education & Innovation Center: skill deficiency in bile duct injury recognition among surgical residents
Source: Surg Endosc. 2025 Sep 13;39(11):7727–35. doi: 10.1007/s00464-025-12145-x (PMC12618438; doi:10.1007/s00464-025-12145-x)
Supplement: Supplementary file 1 — Supplementary file1 (PDF 610 KB) [file 464_2025_12145_MOESM1_ESM.pdf]

APPENDIX A: CHOLANGIOGRAM QUIZ

Project Interpret Cholangiogram: SAGES 2024 Education & Innovation Center Quiz
[Please do not distribute these without expressed permission from the authors. Correct answers are in ***bold.****]* * †

Question 1: Interpret the following cholangiogram.

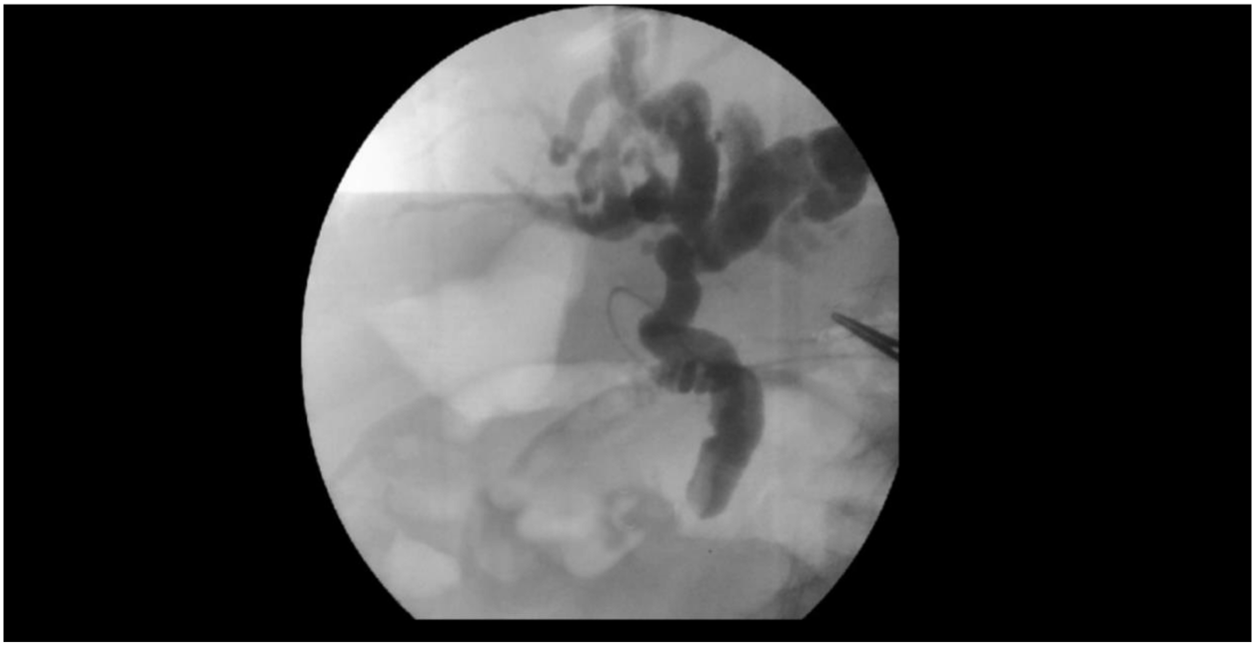

1. Normal intraoperative cholangiogram
2. **CBD lesion**
3. CBD Calculus
4. Common hepatic duct calculus
5. Pancreatic duct filling defect
6. Technical error in shooting cholangiogram

Question 2: Interpret the following cholangiogram.

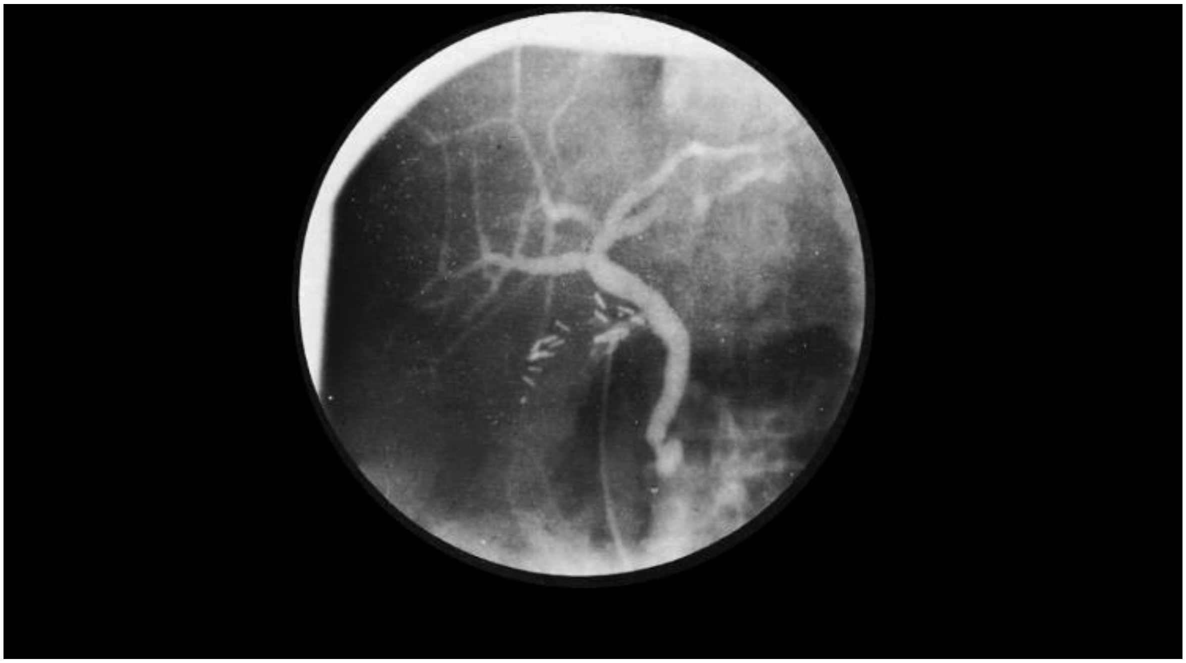

1. Additional cystic duct entering CBD
2. Dilation of CBD
3. Low union of hepatic duct with cystic duct
4. Parallel run of cystic duct with common bile duct
5. **Diverticula arising from CBD**
6. Normal intraoperative cholangiogram

Question 3: Interpret the following cholangiogram.

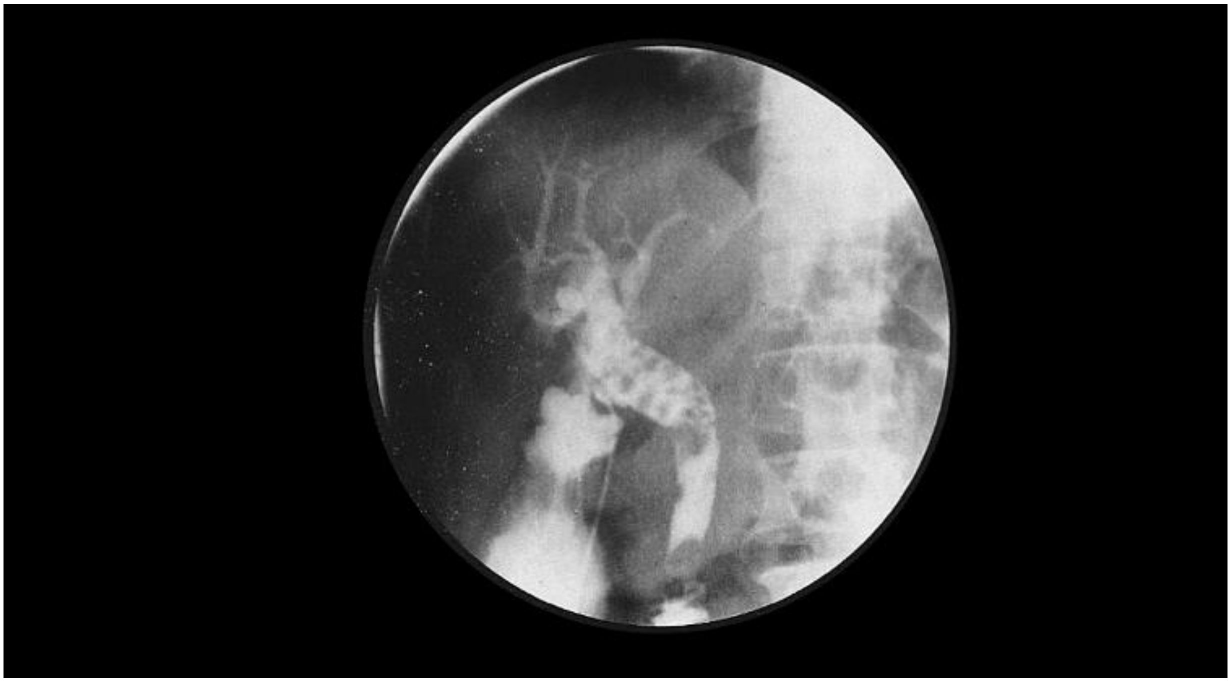

1. Normal intraoperative cholangiogram
2. Cystic duct calculi
3. **Common bile duct calculi**
4. Common hepatic duct calculi
5. Hepatic duct filling defect
6. Pancreatic duct filling defect

Question 4. Interpret the following cholangiogram.

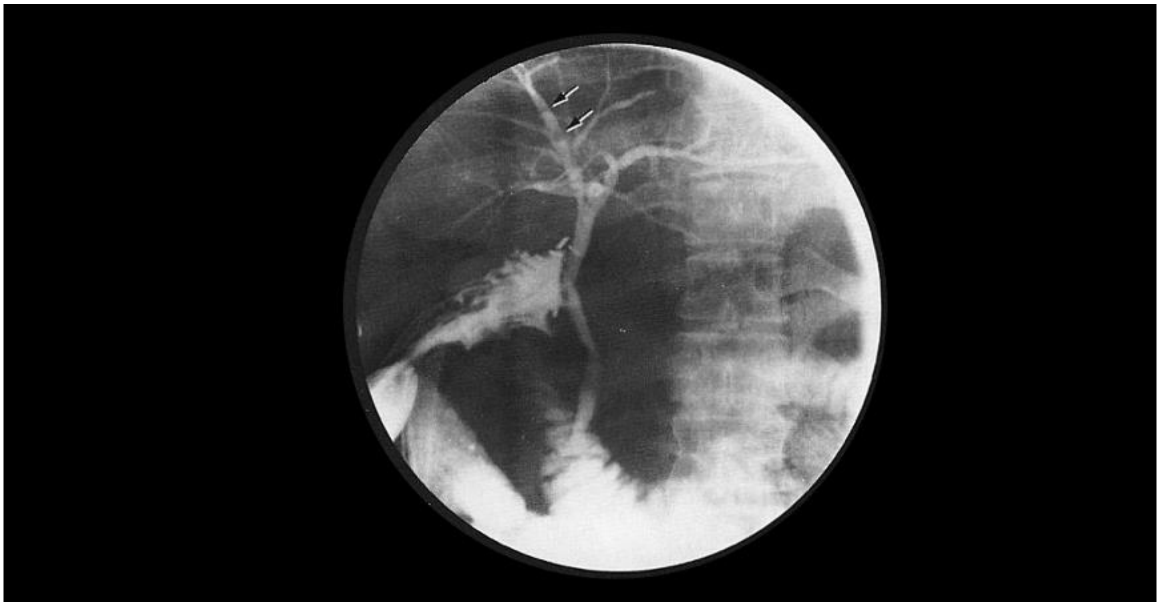

1. Normal intraoperative cholangiogram
2. Cystic duct filling defect
3. Dilation of common bile duct
4. **Hepatic duct filling defect**
5. Pancreatic duct filling defect
6. Technical error in shooting cholangiogram

Question 5. Identify the anatomic anomaly in the biliary tree.

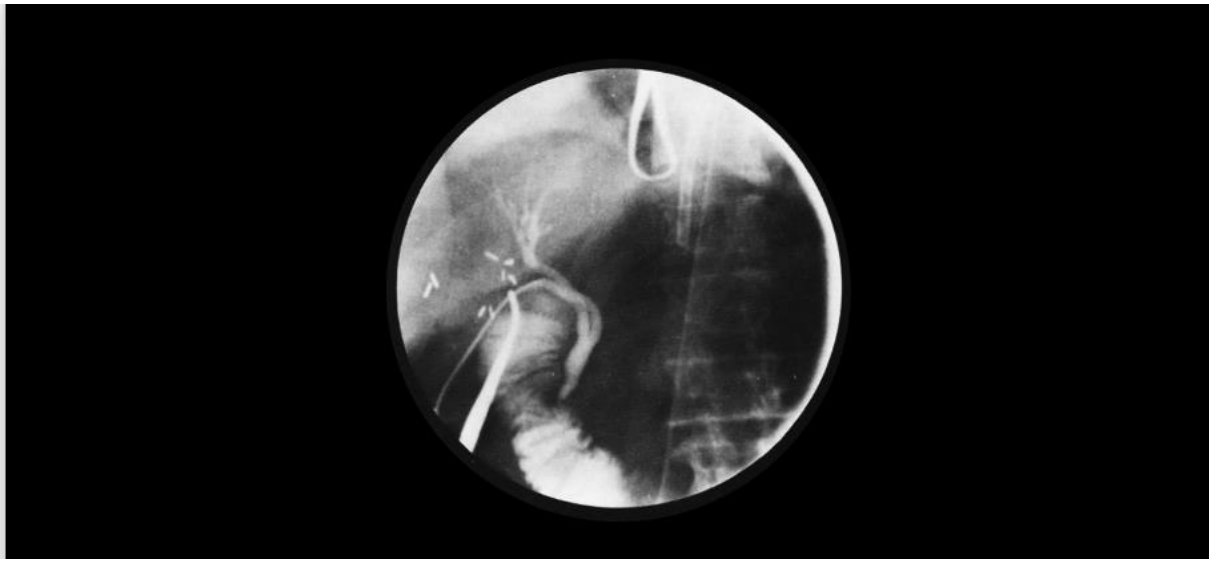

1. Additional cystic duct entering CBD
2. Additional right hepatic duct entering CBD
3. **Spiral entry of cystic duct**
4. Absent left hepatic duct
5. Union of cystic duct and right hepatic duct before entering CBD
6. Normal intraoperative cholangiogram

Question 6. Interpret the following cholangiogram.

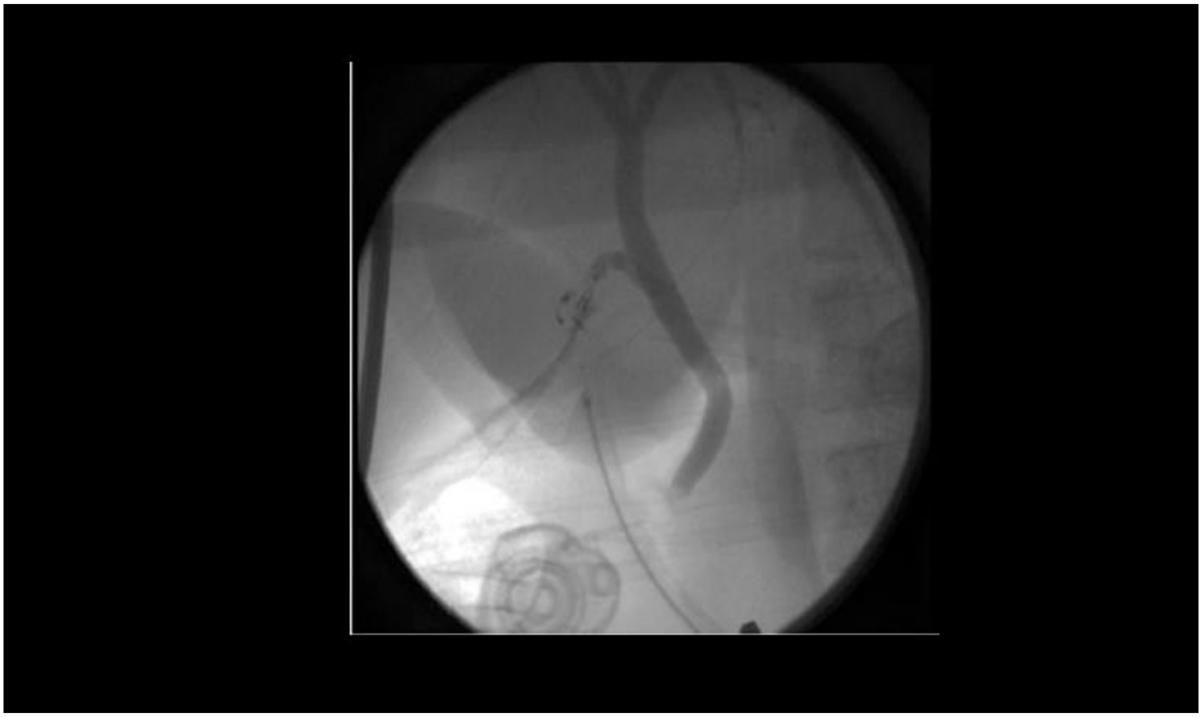

1. Normal intraoperative cholangiogram
2. Cystic duct calculus
3. **Common bile duct calculus**
4. Common hepatic duct calculus
5. Malignant obstruction of CBD
6. Pancreatic duct filling defect

Question 7. Interpret the following cholangiogram.

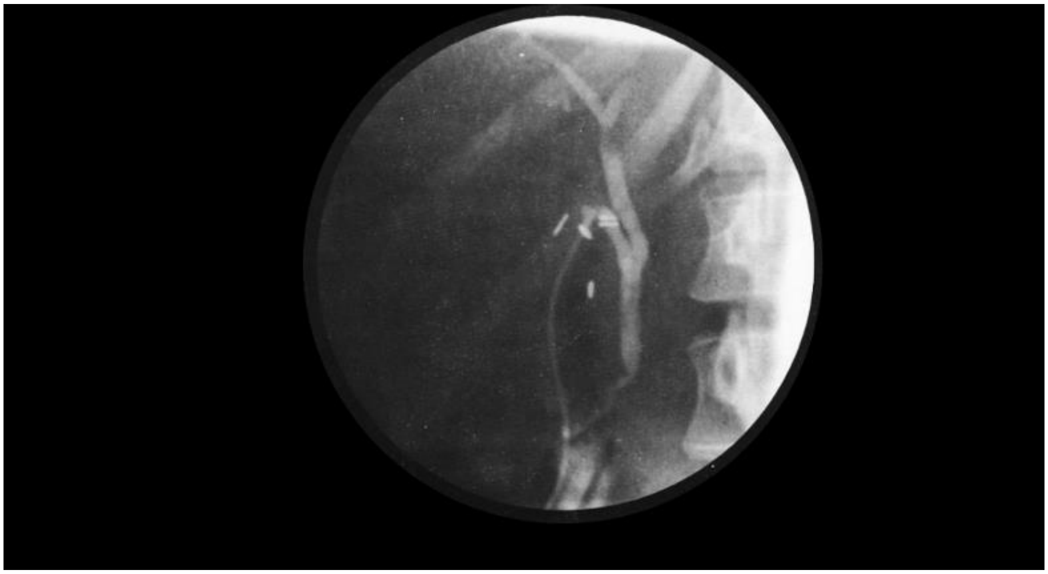

1. Normal intraoperative cholangiogram
2. Cholelithiasis
3. Choledocholithiasis
4. Low union of hepatic duct with cystic duct
5. Spiral entry of cystic duct
6. **Parallel run of cystic duct with common bile duct**

Question 8. Interpret the following cholangiogram.

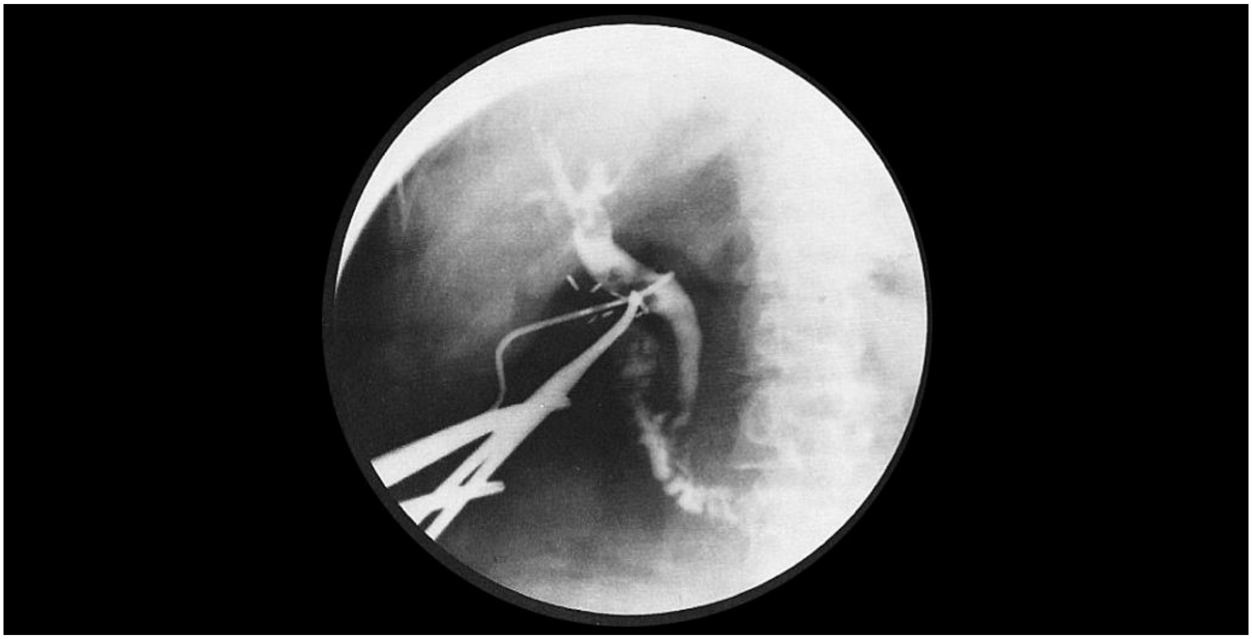

1. **Choledocholithiasis with dilation of common hepatic duct**
2. Choledocholithiasis without dilation of common hepatic duct
3. Normal intraoperative cholangiogram
4. Pancreatic duct filling defect
5. Malignant obstruction of CBD
6. Technical error in shooting cholangiogram

Question 9. Interpret the following cholangiogram.

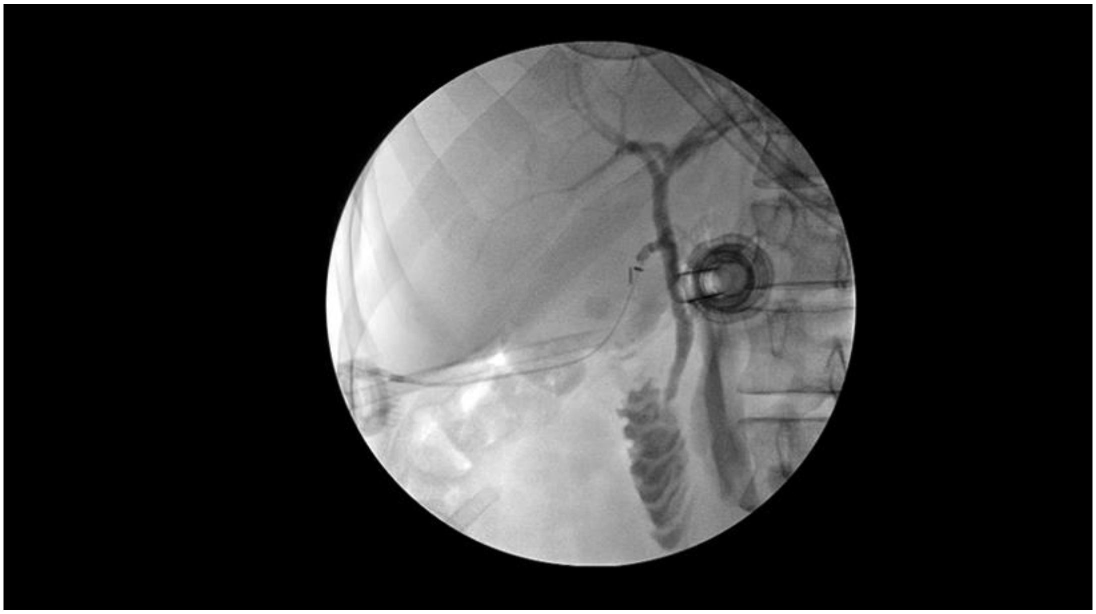

1. **Normal intraoperative cholangiogram**
2. Cystic duct calculus
3. Common bile duct calculus
4. Common hepatic duct calculus
5. Malignant obstruction of CBD
6. Pancreatic duct filling defect

Question 10. Interpret the following cholangiogram.

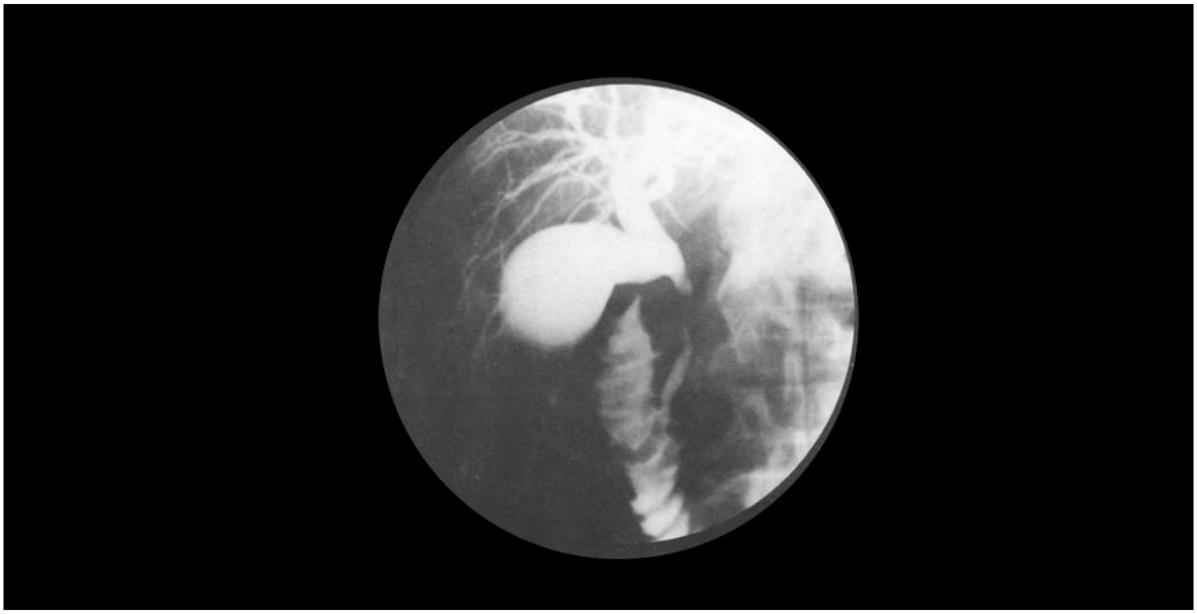

1. Extravasation of contrast material
2. **Malignant obstruction of CBD**
3. Cholelithiasis
4. Choledocholithiasis
5. Normal intraoperative cholangiogram
6. Technical error in shooting cholangiogram

Question 11. Identify the anatomic anomaly in the biliary tree.

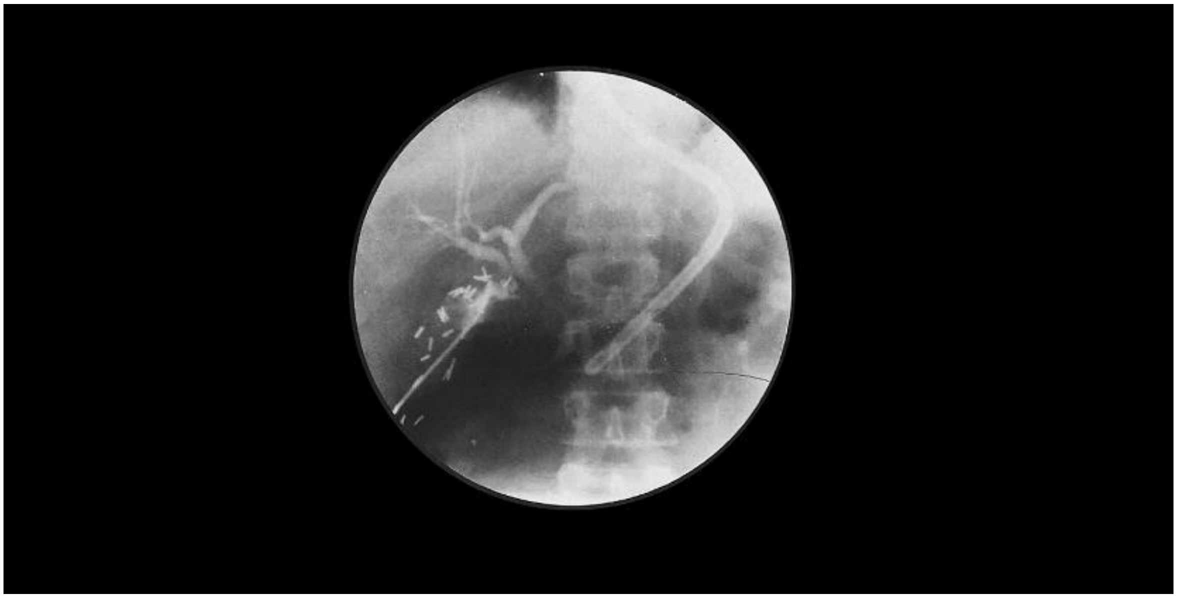

1. Additional cystic duct entering CBD
2. Additional right hepatic duct entering CBD
3. Spiral entry of cystic duct
4. Absent left hepatic duct
5. **Union of cystic duct and right hepatic duct before entering CBD**
6. Normal intraoperative cholangiogram

Question 12. Interpret the following cholangiogram.

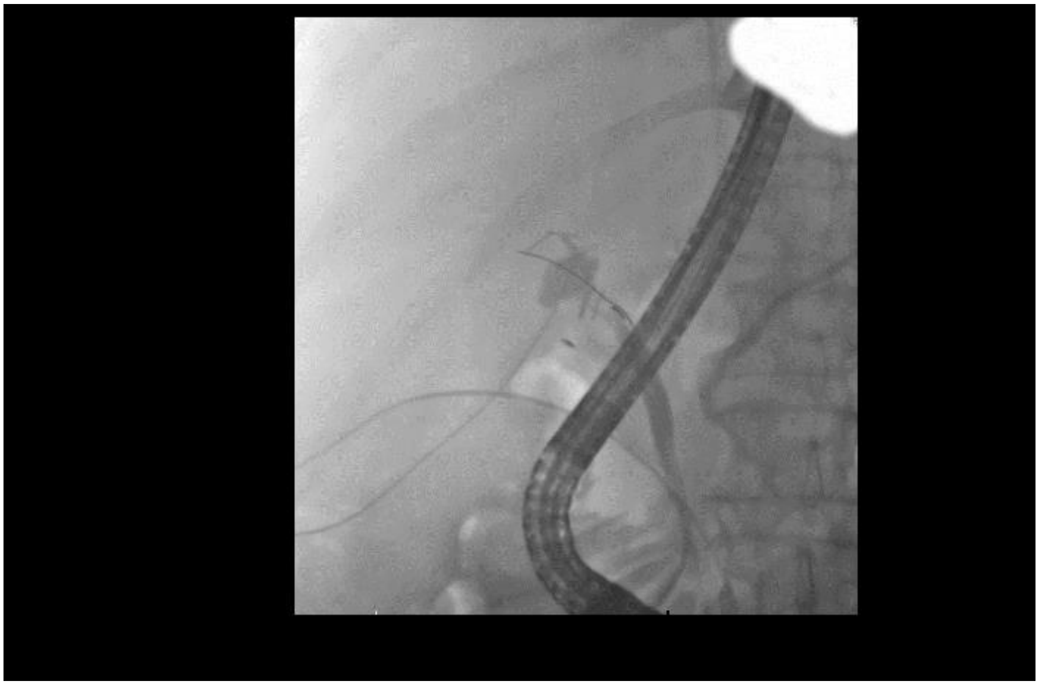

1. Normal intraoperative cholangiogram
2. Technical error in shooting cholangiogram
3. Calculus in CBD
4. Dilated CBD
5. **CBD injury**
6. Spiral entry of cystic duct

Question 13. Interpret the following cholangiogram.

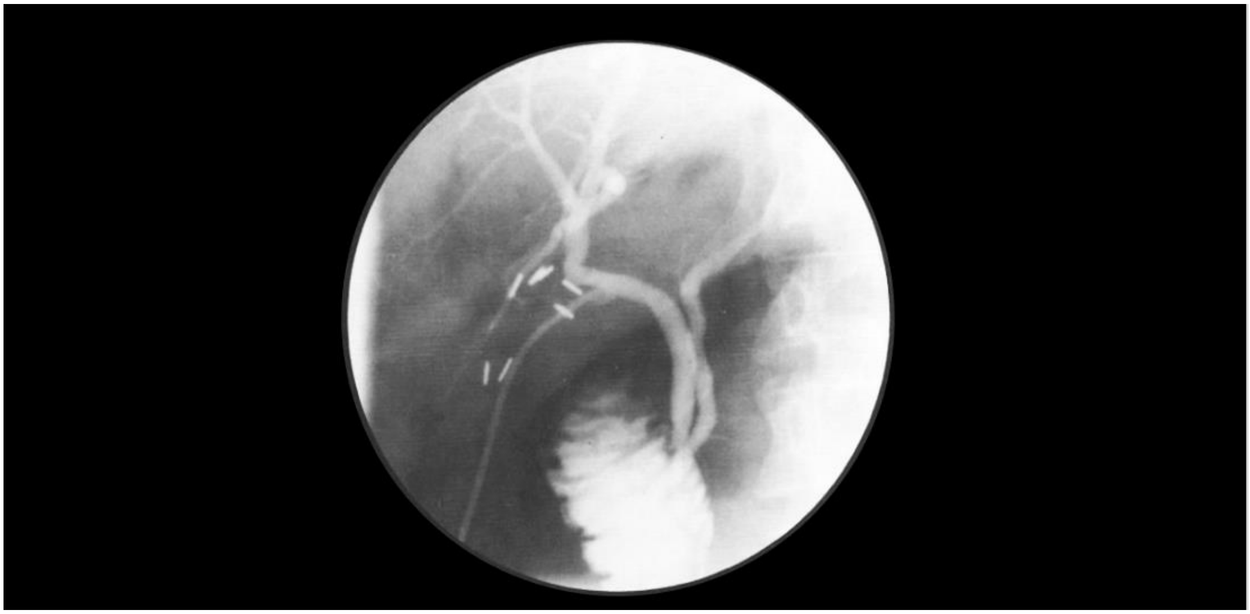

1. **Normal cystic duct cholangiogram**
2. Aberrant right hepatic duct
3. Pancreatic duct filling defect
4. Dilated common bile duct
5. Common bile duct lesion
6. Parallel run of cystic duct with common bile duct

Question 14. Interpret the following cholangiogram.

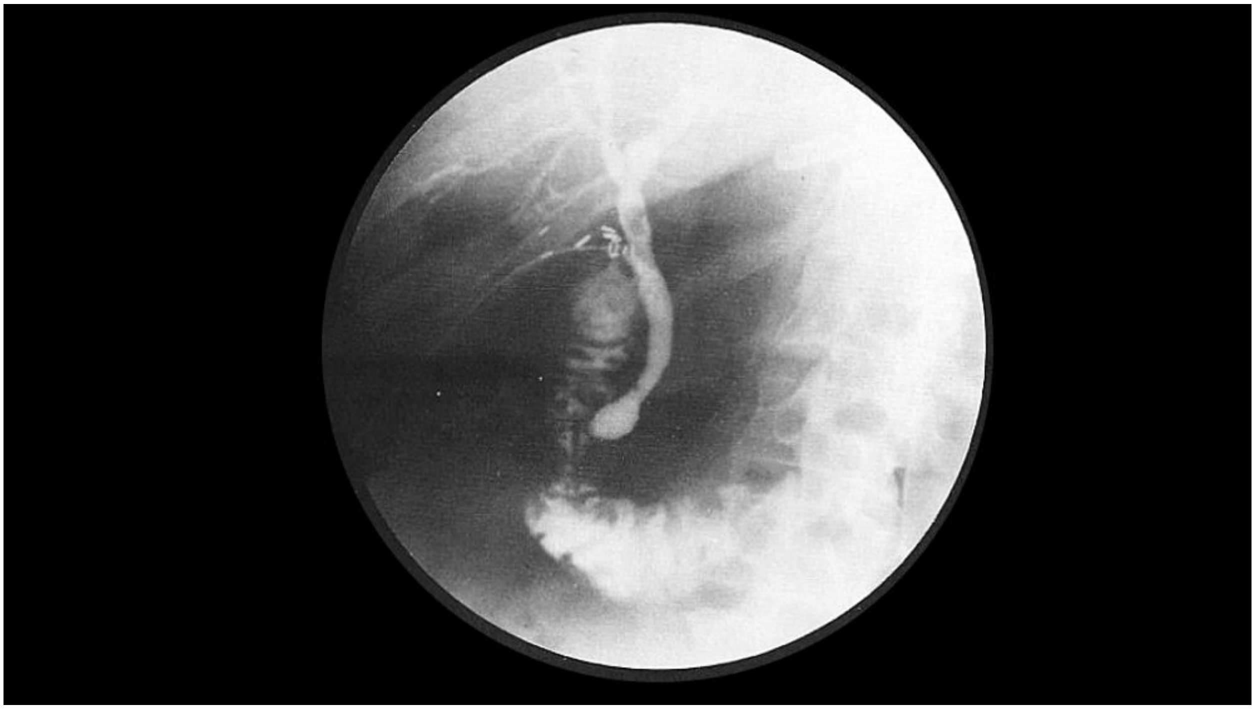

1. Normal intraoperative cholangiogram
2. Cystic duct calculus
3. Common bile duct calculus
4. **Common hepatic duct calculus**
5. Hepatic duct filling defect
6. Pancreatic duct filling defect

Question 15. Interpret the following cholangiogram.

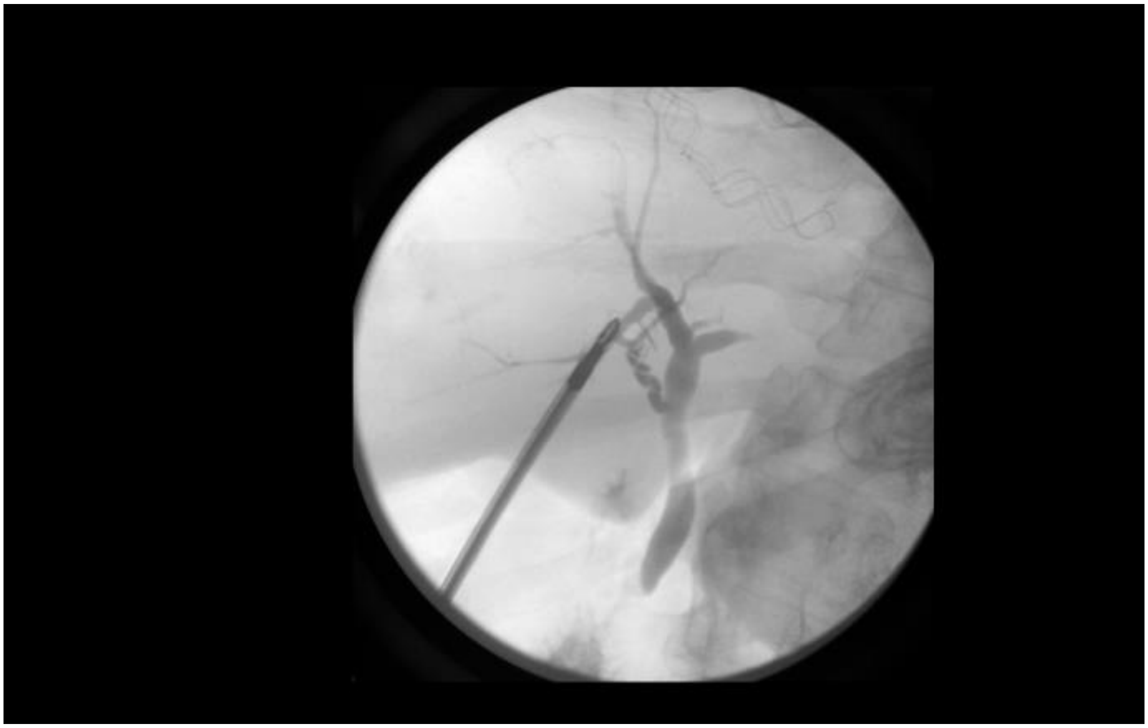

1. Normal intraoperative cholangiogram
2. Cystic duct calculus
3. Common bile duct calculus
4. Common hepatic duct calculus
5. Malignant obstruction of CBD
6. **No flow in duodenum and hepatocystic duct**

Question 16. Interpret the following cholangiogram.

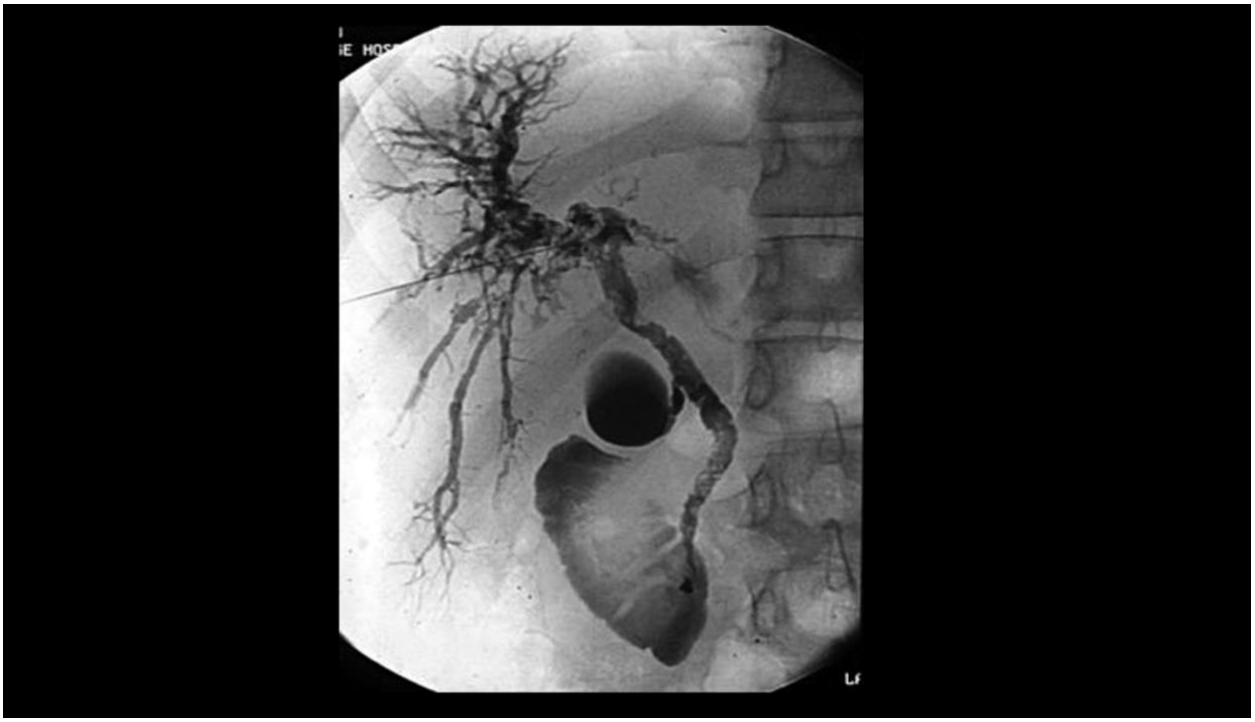

1. Normal intraoperative cholangiogram
2. Malignant obstruction of CBD
3. **Sclerosing cholangitis**
4. Low union of hepatic duct with cystic duct
5. Spiral entry of cystic duct
6. Parallel run of cystic duct with common bile duct

Question 17. Interpret the following cholangiogram.

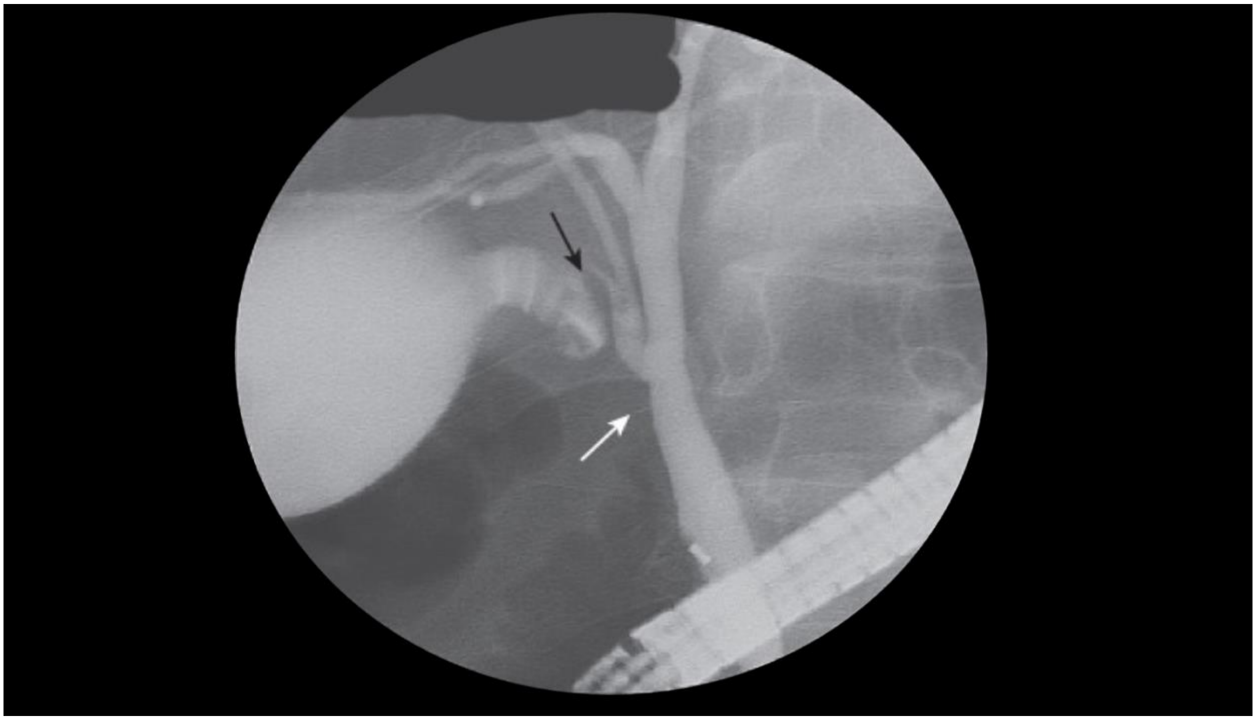

1. **Cystic duct coming off accessory duct**
2. CBD injury
3. Low union of hepatic duct with cystic duct
4. Parallel run of cystic duct with common bile duct
5. Diverticula arising from CBD
6. Normal intraoperative cholangiogram

Question 18. Interpret the following cholangiogram.

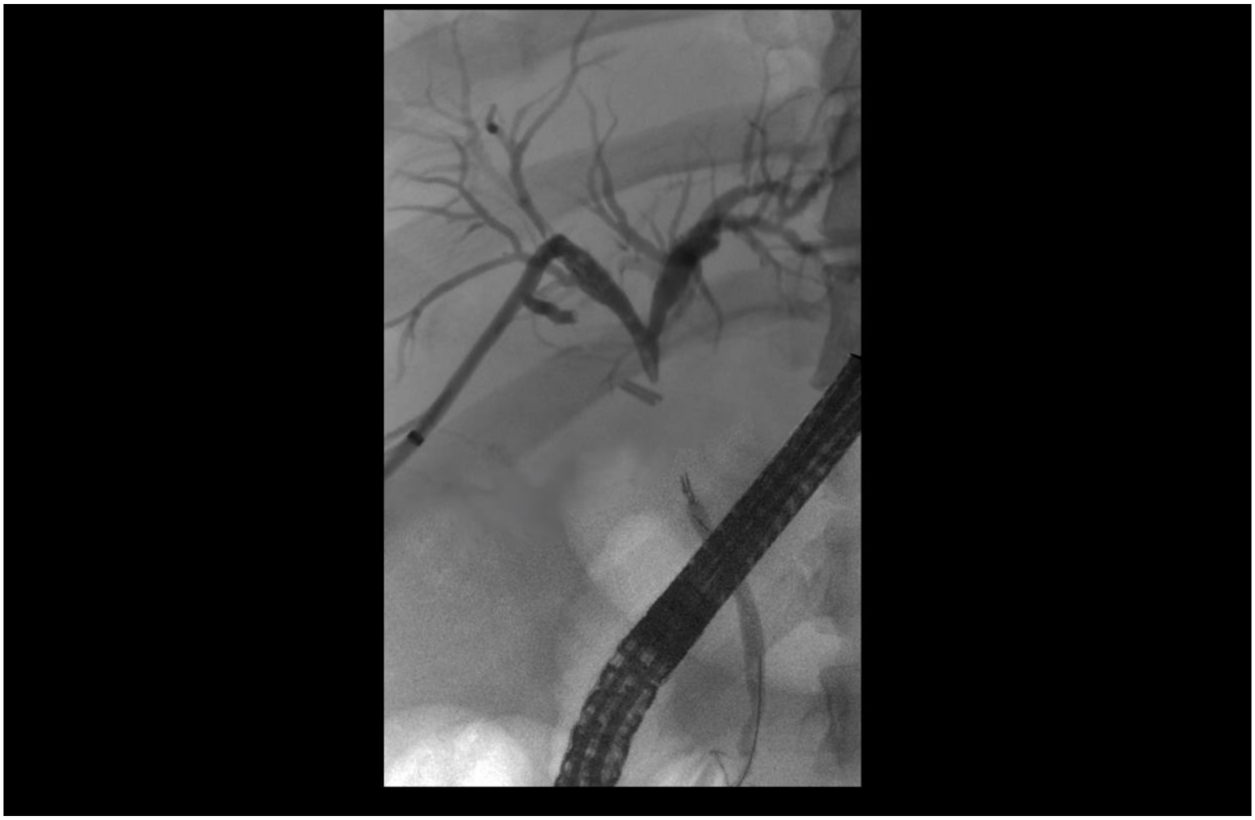

1. Normal intraoperative cholangiogram
2. Low union of hepatic duct with cystic duct
3. Malignant obstruction of CBD
4. Diverticula arising from CBD
5. **CBD injury**
6. Technical error in shooting cholangiogram

[END OF QUIZ]

*Cholangiograms in questions 2-5, 7, 8, 10, 11, 13, 14 adapted from: Berci G, Hamlin JA. *Operative Biliary Radiology*. Williams & Wilkins; 1981.

†Cholangiograms in questions 1, 6, 9, 12, 15-18 reproduced with permission from Dr. Steven D. Schwaitzberg
